# Importance of polyphosphate in the *Leishmania* life cycle

**DOI:** 10.15698/mic2018.08.642

**Published:** 2018-06-22

**Authors:** Kid Kohl, Haroun Zangger, Matteo Rossi, Nathalie Isorce, Lon-Fye Lye, Katherine L. Owens, Stephen M. Beverley, Andreas Mayer, Nicolas Fasel

**Affiliations:** 1 Department of Biochemistry, University of Lausanne, Epalinges, Switzerland.; 2 Department of Molecular Microbiology, Washington University School of Medicine, St. Louis, Missouri, United States of America.

**Keywords:** polyphosphate, VTC4, Leishmania, life cycle, infectivity, temperature stress

## Abstract

Protozoan parasites contain negatively charged polymers of a few up to several hundreds of phosphate residues. In other organisms, these poly-phosphate (polyP) chains serve as an energy source and phosphate reservoir, and have been implicated in adaptation to stress and virulence of pathogenic organisms. In this study, we confirmed first that the polyP polymerase vacuolar transporter chaperone 4 (*VTC4*) is responsible for polyP synthesis in *Leishmania *parasites. During *Leishmania* *in vitro *culture, polyP is accumulated in logarithmic growth phase and subsequently consumed once stationary phase is reached. However, polyP is not essential since VTC4-deficient (*vtc4^-^*) *Leishmania* proliferated normally in culture and differentiated into infective metacyclic parasites and into intracellular and axenic amastigotes. In *in vivo *mouse infections, *L. major* *VTC4 *knockout showed a delay in lesion formation but ultimately gave rise to strong pathology, although we were unable to restore virulence by complementation to confirm this phenotype. Knockdown of *VTC4* did not alter the course of *L. guyanensis* infections in mice, suggesting that polyP was not required for infection, or that very low levels of it suffice for lesion development. At higher temperatures, *Leishmania* promastigotes highly consumed polyP, and both knockdown or deletion of *VTC4* diminished parasite survival. Thus, although polyP was not essential in the life cycle of the parasite, our data suggests a role for polyP in increasing parasite survival at higher temperatures, a situation faced by the parasite when transmitted to humans.

## INTRODUCTION

*Leishmania *parasites can cause leishmaniasis, a disease affecting over 12 million people in 98 countries in tropical and subtropical regions 1 and are responsible for different immuno-pathologies: cutaneous, mucosal and visceral, depending mainly on the infecting species. As part of their life cycle, *Leishmania *need to switch between two different forms, which allow them to better adapt to each host. These protozoan parasites live either as intracellular amastigotes in the phagolysosome of human macrophages or as extracellular flagellated promastigotes in different sand fly species (e.g. *Phlebotomus* or *Lutzomyia*). In their insect vector, after a blood meal, *Leishmania* differentiate from amastigotes into proliferating procyclic promastigotes, which further differentiate into stationary infectious metacyclic promastigotes 2. While cycling between two hosts, the parasites are exposed to major environmental changes such as pH, temperature, reactive oxygen species, nutrient and oxygen availabilities. Polymers of inorganic phosphate (polyP) might play a role in adaptation to these drastic changes of environment as they have been implicated in stress tolerance in several other organisms 3.

Many bacterial, archaeal, fungal, protozoan, plant or animal organisms contain polyP. These are linear polymers of inorganic phosphate composed of a few up to many hundreds of orthophosphate residues linked by phosphoric anhydride bonds. Despite their prevalence, polyPs are understudied and they have long been dismissed as ‘molecular fossils’ which serve as a source of phosphate and energy. However, we now begin to understand that polyP is an active element of metabolism that contributes to adaptations to stress and also to virulence. In prokaryotes, for instance, polyP synthesis is crucial for *Escherichia coli* transition from active growth into stationary phase and for cell survival under nutrient starvation, heat, oxidative or osmotic stress and UV irradiation (reviewed in 3). PolyP also influences bacterial gene transcription, motility, quorum sensing, virulence and the formation of ion channels and biofilms (reviewed in 4,5,6,7). In the absence of the polyP kinase 1 (PPK1), some bacterial pathogens display growth disorders, loss of viability, intolerance to acid and heat and diminished invasiveness into epithelial cells 3,8,9, some of which may be related to the capacity of polyP to chaperone proteins 10. In mammals, the presence of polyP chains has been discovered much later, probably due to its lower concentration. PolyP functions in mammals have been related to cancer 1,11, apoptosis 12, bone mineralization 13, osteoblast function 14, fibroblast growth stimulation 15 and energy metabolism 16, to which much attention has been drawn to lately for their pro-coagulant and pro-inflammatory effects 17,18.

In bacteria, polyP is mainly synthesized by two polyP kinases, PPK1 and PPK2. PPK1 is responsible for the major portion of polyP in the cell as it catalyzes the transfer of the gamma-phosphate of ATP to the nascent polyP chain. Even though this reaction is reversible it favors the synthesis of polyP. PPK2 can synthesize polyP from GTP but it favors the reverse reaction of polyP degradation 19,20. No homologs of the PPKs could be found in eukaryotes, except for the slime mold *Dictyostelium discoideum*. In the yeast *Saccharomyces cerevisiae*, a large number of genes are required for the synthesis of polyP 21,22, among which is a polyP polymerase, named vacuolar transporter chaperone 4 (Vtc4), which represents the first eukaryotic-specific polyP synthase described 23. Vtc4 is a transmembrane protein that forms different complexes with other Vtc proteins, either in the combination of Vtc1, Vtc2 and Vtc4 or Vtc1, Vtc3 and Vtc4 23,24,25,26. These Vtc complexes synthesize polyP from nucleotide triphosphates and translocate it at the same time into the acidocalcisome-like vacuoles, where polyP is sequestered in order to avoid its toxic effects in the cytosol. Moreover, Vtc complexes can be stimulated by association with a fifth subunit, Vtc5, or by binding of inositol pyrophosphates to their SPX domains 27,28,29. In yeast, Vtc deficiency affects the ability of the cell to grow under phosphate-limiting or metal-limiting conditions 23,30, mildly reduces vacuolar proton pump function and influences organelle biogenesis through cargo traffic between the ER and Golgi, vacuolar fusion and micro-autophagy 24,25,31. As the polyP polymerases in higher eukaryotes are still unknown, it remains difficult to experimentally interfere with polyP levels and explore polyP functions in animals and plants.

In trypanosomatid parasites, such as *Trypanosoma *or *Leishmania,* polyP is highly concentrated in acidocalcisomes, which are acidic organelles that besides the negatively charged polyP, also contain cations (Ca^2+^, Mg^2+^, Na^+^, K^+^, Zn^2+^, Fe^2+^) and basic amino acids (mainly arginine and lysine) 32,33,34,35,36,37,38,39. Accumulating data suggests that polyP and acidocalcisomes in parasites play an important role for adaptation to stresses and affect disease outcome 32,33,35,36,40,41,42,43,44,45. Although several studies have been performed on acidocalcisomes in *Trypanosoma*, little is known about polyP expression and function in *Leishmania*. In this study, we investigated whether polyP plays a role in the life cycle of *Leishmania* parasites and in its survival under stress conditions.

## RESULTS

### Identification of the *VTC4* gene in *Leishmania*

**Figure 1:**
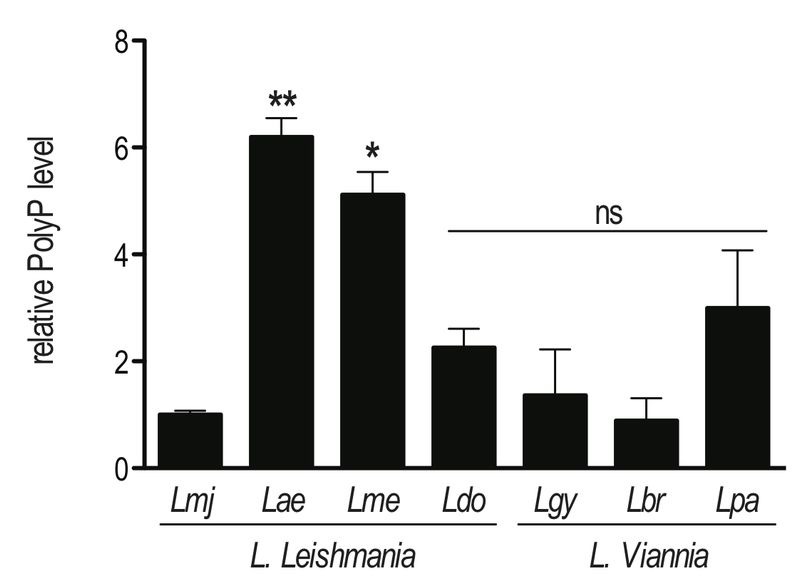
FIGURE 1: High variability of polyP abundance in different *Leishmania* species. Parasites were grown in complete Schneider’s medium. PolyP was extracted from 2 x 10^7^ logarithmic phase (day 3 post dilution) promastigotes, digested with the polyphosphatase Ppx1, and released P_i_ was quantified colorimetrically. Results of a pool of 2 independent experiments were expressed as mean ± SD of polyP level relative to *L. major*. Statistical significance was assessed by *Student’s t-test*; * p < 0.05, ** p < 0.01, ns: non-significant. *Lmj, L. major *(IR75);* Lae, L. aethiopica *(LDS372);* Lme, L. mexicana *(M379);* Ldo, L. donovani *(AG83);* Lgy, L. guyanensis *(M5313);* Lbr, L. braziliensis *(LTB325); *Lpa, L. panamensis *(1166).

In order to investigate the role of polyP in the life cycle of *Leishmania*, we first identified the enzyme responsible for polyP synthesis and confirmed its presence in different *Leishmania* species. Knowing that VTC4 is responsible for polyP synthesis in yeast and *T. brucei*, we searched for vtc4 homologues in *L. major*. Database mining of the *L. major* genome for yeast or *T. brucei* *VTC* homologues revealed a single gene coding for VTC4 localized on chromosome 9 (LmjF09.0220). The *LmjVTC4* open reading frame (ORF) encodes an 813 amino acid protein with a predicted molecular mass of 93.4 kDa. Using this information, we also identified *VTC4* in other *Leishmania* species and cloned it from the *L. guyanensis* genome (GenBank access number: MF572933). Using alignments by ClustalW within Jalview 46,47, the *Lmj*VTC4 amino acid sequence appears quite conserved with respect to VTC4 from other *Leishmania* and *Trypanosoma* species: 97% sequence identity with *L. infantum*, 95% with *L. mexicana*, 88% with *L. braziliensis* and *L. guyanensis*, and 67% with *T. brucei* and *T. cruzi* (Fig. S1). We also used an enzymatic assay to test for the presence of polyP in different *Leishmania* species of two distinct subgenera. Using this assay, we confirmed that polyP was present in every *Leishmania* species analyzed, albeit at significantly different abundance (Fig. 1). However, we could not correlate the different levels of polyP to a specific *Leishmania* subgenus (*L. Leishmania* or *L. Viannia*).

### Decreased mRNA and polyP levels in *VTC4* knockdown *L. guyanensis* parasites

**Figure 2:**
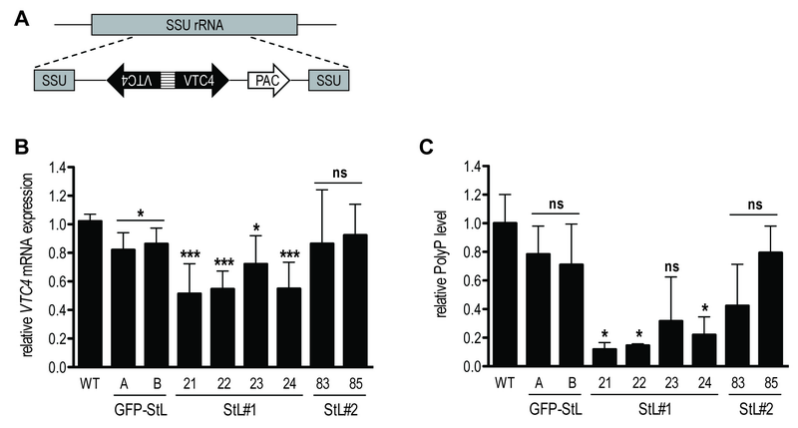
FIGURE 2: RNA interference on *VTC4* in *L. guyanensis*. **(A)** Schematic map of SSU rRNA locus in *Leishmania* and targeting of *VTC4*. Regions are the SSU rRNA (grey box), *VTC4* region (black arrow), puromycin-resistance gene ORF (*PAC*, white arrow) and the stem loop stuffer fragment (striped box). **(B)** Relative *VTC4* mRNA levels were assessed by qRT-PCR with *VTC4* and *KMP11 *gene primers. Primer sequences are presented in Table S2. **(C)** Relative polyP was quantified by digesting the extracted polyP and measuring the released Pi colorimetrically. A and B; 21, 22, 23 and 24; 83 and 85 represented recombinant *Leishmania* clones selected by antibiotic resistances, for more detail see Fig. S2. Results of a pool of 3 independent experiments were expressed as mean ± SD. Statistical significance was assessed by *Student’s t-test* **(B **and** C)**; * p < 0.05, *** p < 0.001, ns: non-significant.

To confirm the relation between VTC4 and polyP synthesis in *Leishmania* parasites, *VTC4* was knocked down in *L. guyanensis,* taking advantage of the presence of the RNA degradation machinery in the *Leishmania *(*Viannia*) subgenus 48,49. We used the RNA interference (RNAi) approach to generate *VTC4* knockdown *L. guyanensis* in a single round of transfection. For this purpose, two stem-loop (hairpin) RNAi constructs were generated using the pIR integrating transfection vectors that target either a region at the beginning (193 - 788, referred to as StL#1) or at the center (867 - 1963, referred to as StL#2) of the *L. guyanensis* *VTC4* ORF (Fig. S2). These stem loop constructs contained two copies of the targeted region in an inverted orientation separated by a short loop (Fig. 2A), and were flanked by *Leishmania* sequences required for efficient 5’ and 3’ end mRNA formation, as well as a selectable drug resistance gene 48. Upon parasite transfection, the linearized DNA was integrated into the small subunit ribosomal RNA (SSU) locus. As a control, parasites with a *GFP* targeting stem loop (*GFP*-StL) were generated 48,50. Clones were selected due to their drug resistance and DNA integration was confirmed by PCR (Fig. S2B, C).

In order to investigate the knockdown efficiency, we performed a quantitative real time (qRT-) PCR on *VTC4* transcripts 50. Relative expression of *VTC4* mRNA levels were significantly decreased in StL#1 transfected clones, while there was no decrease in StL#2 transfectants (Fig. 2B). Although Lye *et al.* observed more than 10-fold mRNA reduction in their Luciferase and *GFP* knockdowns 48, we detected a less than 2-fold reduction for StL#1 in the same strain of *L. guyanensis*. qRT-PCR can, however, detect RNA degradation products which can accumulate even though they are non-functional and thus underestimate the effect on the protein level 48,51.

We measured presumptive VTC4 activity by extracting and quantifying polyP of logarithmic phase RNAi transfectants. The polyP levels of StL#1 transfectants were decreased three- to five-fold, whereas the effects in StL#2 and in *GFP*-StL transfectant controls were not statistically significant (Fig. 2C), thus confirming the qRT-PCR data (Fig. 2B). As variability in efficiency amongst RNAi constructs is not uncommon, for subsequent studies we used only the two StL#1 transfectants (clones 21 and 22) showing the strongest knockdown.

### *vtc4^-^ L. major* is devoid of short-chain polyP, displays normal promastigote growth and differentiates into metacyclic promastigotes

**Figure 3:**
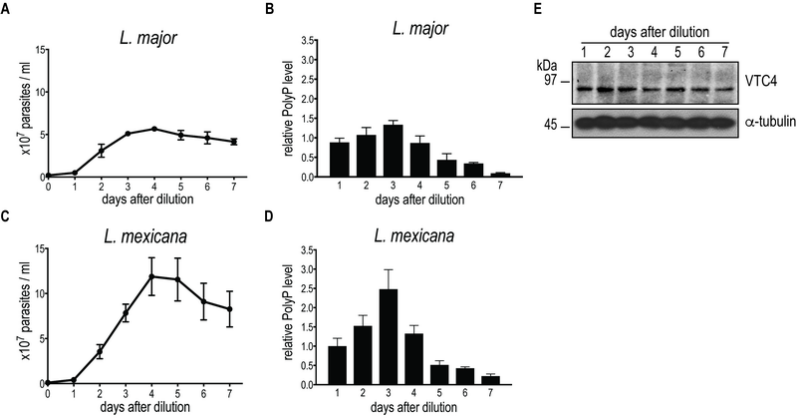
FIGURE 3: PolyP and VTC4 fluctuation during promastigote growth of *L. major *and *L. mexicana. * *L. major* **(A) **and *L. mexicana* **(C) **promastigote cell concentrations were counted in independent cultures. **(B, D)** PolyP was extracted from cell lines, digested with polyphosphatase and P_i_ were quantified colorimetrically using malachite green. P_i_ concentrations represented polyP content of 3 x 10^7^ cells for *L. major ***(B) **and 8 x 10^6^ cells for *L. mexicana ***(D)***.* Results of a pool of minimum 3 independent experiments were expressed as mean ± SD. **(E)** Western blot analysis of 20 µg of *L. major *promastigotes pellet protein using the anti-cd-*Lmj*VTC4 antibody and anti-α-tubulin as loading control.

We first measured polyP levels and lengths during the *in vitro* transition of *L. major* and *L. mexicana* promastigotes from logarithmic into stationary phase (Fig. 3) using a protocol detecting mainly short-chain polyP (< 300 residues). From a stationary phase culture, parasites were diluted to a concentration of 5 x 10^5^ parasites/ml, cultured over 7 days and cell density was measured every day (Fig. 3A, C). In addition, polyP was extracted, digested and quantified at each time point. In promastigotes of both *Leishmania* species tested, polyP was most abundant in late logarithmic growth phase promastigotes (day 3 post dilution), while it gradually decreased overtime in long term stationary phase cultures (day 4 to 7 post dilution) (Fig. 3B, D), suggesting that polyP synthesis occurred mainly in proliferating parasites, and consumed when stationary parasites were maintained in culture for a long period. To test whether VTC4 was still expressed in stationary phase parasites, and to confirm its absence in *vtc4^-^* parasites in parallel, we used polyclonal antibodies against the *L. major* protein for use in western blotting 30. The central domain (a.a. 202 - 504) of *Lmj*VTC4 (cd-*Lmj*VTC4) was expressed in bacteria and used for rabbit immunization. In contrast to polyP, VTC4 levels stayed constant during the entire promastigote stage, suggesting that VTC4 might be inactivated by post-translational regulation, or the degradation of polyP chains might be increased during stationary phase (Fig. 3E). As controls for our antibody, we previously used *vtc4^-^* parasites and confirmed the absence of *VTC4* *in vtc4^-^* parasites 30.

**Figure 4:**
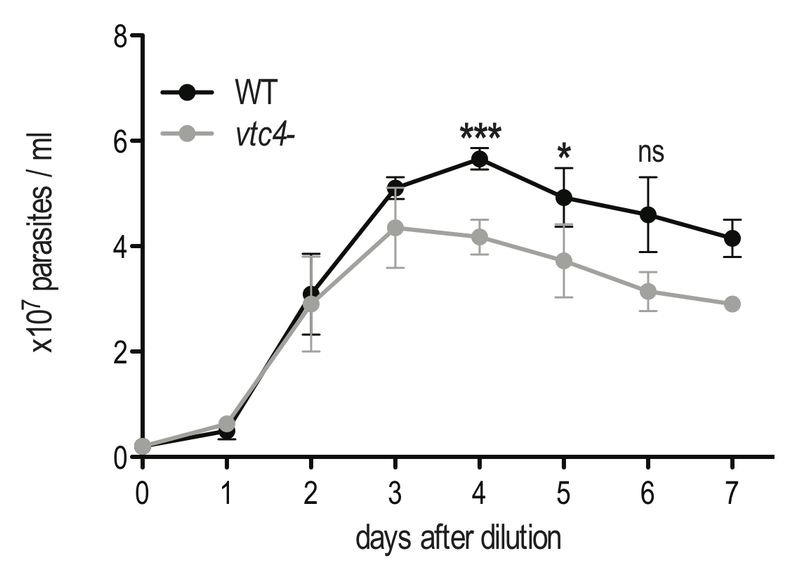
FIGURE 4: *In vitro *proliferation of *VTC4* knockout *L. major *parasites. *L. major* WT or *vtc4^-^* promastigotes were diluted at a concentration of 5 x 10^5^ parasites per ml and cultured in complete medium. Cell concentrations were counted in independent cultures. Results of a pool of 4 independent experiments were expressed as mean ± SD. Statistical significance was assessed by *Student’s t-test*; * p < 0.05, *** p < 0.01, ns: non-significant.

Although VTC4 was essential for polyP synthesis in *Leishmania*, and apparently no other enzyme complemented its function, *vtc4^-^* promastigotes had a morphology similar to WT and proliferated in culture similarly to WT promastigotes, demonstrating that VTC4 was not essential for promastigote viability and proliferation *in vitro*. However, WT and *vtc4*^-^ clones did not reach the same cell density in culture (Fig. 4) suggesting that VTC4 could be important for parasite survival in nutrient-limited conditions*.*

**Figure 5:**
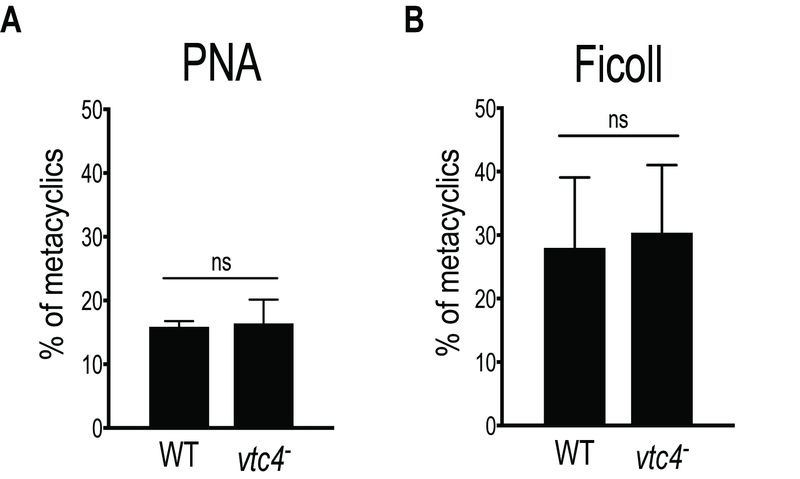
FIGURE 5: Normal metacyclogenesis in absence of polyP in *L. major*. Metacyclic promastigotes of WT and *vtc4^-^ L. major *clones were isolated by PNA assay **(A)** or by Ficoll centrifugation gradient **(B)**. Results of a pool of 2 independent experiments were expressed as mean ± SD. Statistical significance was assessed by *Student’s t-test* **(A **and** B)**; ns: non-significant.

Since polyP is important in the transition from active growth into stationary phase in bacteria 3 and play a role in parasite virulence 32,36,52,53, we explored whether *vtc4^-^* parasites were able to differentiate into infectious metacyclic promastigotes. In *L. major*, metacyclogenesis is accompanied by changes in parasite morphology, size and in the composition of the parasite glycocalyx, including lipophosphoglycan (LPG) 54,55. To evaluate the efficiency of metacyclogenesis, stationary promastigotes were fractionated by applying two different methods: a peanut agglutinin (PNA) assay, in which only procyclic LPG binds to lectins 56, and a LPG-independent Ficoll gradient centrifugation method 57. Both methods showed no significant difference in the percentage of metacyclics between *vtc4^-^* and WT parasites (Fig. 5A, B), suggesting that polyP is not required for *Leishmania* development into metacyclic stationary promastigotes.

### Absence of VTC4 expression and polyP in the amastigote stage

**Figure 6:**
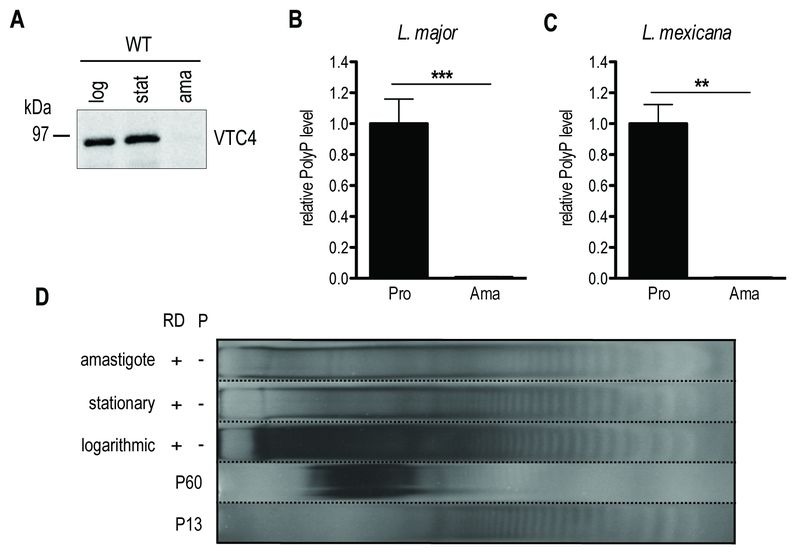
FIGURE 6: VTC4 and polyP levels at different life cycle stages of *L. major* and *L. mexicana*. **(A**) VTC4 abundance in 20 µg of pellet protein at different *L. major* life cycle stages, detected by cd-*Lmj*VTC4 antibody. **(B** and **C)** Relative polyP quantification in *L. major* and *L. mexicana* promastigotes and amastigotes*, *by staining P_i_ residues after polyP digestion. Results of a pool of 2 independent experiments were expressed as mean ± SD. Statistical significance was assessed by *Student’s t-test* **(B **and** C)**; ** p < 0.01, *** p < 0.001. **(D)** PolyP gel displaying polyP abundance in 6 x 10^7^ logarithmic and stationary promastigotes as well as macrophage isolated *L. major *amastigotes. Chains were separated by electrophoresis on a 35% polyacrylamide gel and visualized by negative DAPI staining. log, logarithmic; stat, stationary; ama, amastigotes; pro, promastigotes; RD, RNase/DNase; P, Polyphosphatase. PolyP standards represent an average of the respective sizes ranging from 13 up to 60 residues (P13 and P60).

Subsequently, we determined whether VTC4 was expressed in the amastigote stage by immunoblotting using an anti-VTC4 antibody. VTC4 was almost completely absent from amastigotes isolated from mouse footpad lesions (Fig. 6A). Accordingly, *L. major *amastigotes isolated from lesions, as well as *L. mexicana* axenic amastigotes obtained after 5 days of *in vitro* differentiation contained very low amounts of polyP in comparison to proliferating promastigotes (Fig. 6B and C). We confirmed this result by PAGE of polyP chains from logarithmic growth phase (day 3 post dilution) or stationary phase (day 6 post dilution) promastigotes, and from *L. major* amastigotes isolated from macrophages, visualizing polyP abundance by negative DAPI staining. Polyphosphates were present in relatively high amounts in day 3 logarithmic promastigotes but almost absent in day 6 stationary phase promastigotes and in amastigotes, where mainly short-chain polyphosphates were detectable (Fig 6D). *Vtc4^-^* cells contained neither short nor long-chain polyP, suggesting that LmVtc4 may be the sole enzyme synthesizing polyP in this organism, at least under the growth conditions tested here. However, we cannot exclude the presence of much longer chains, which might exist 58 but may have escaped our assay.

Taken together, these data show that long-chain polyP accumulated during the proliferating logarithmic phase but decreased during long term culture of stationary parasites. Long-chain polyP was almost absent from amastigotes, which however, had similar amounts of shorter-chain polyP as the promastigotes. These data suggest that polyP could be important in the first days for parasites to replicate and to establish an efficient infection as shown previously 30 but not for the differentiation into amastigotes and the development of footpad lesions.

### Relevance of VTC4 in infections *in vivo*

**Figure 7:**
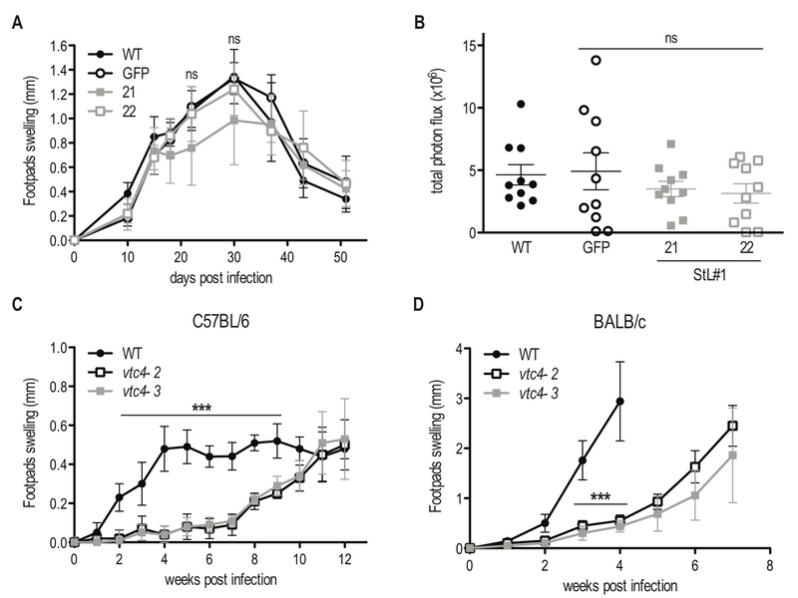
FIGURE 7: *In vivo* infection with *VTC4* knockdown and knockout *Leishmania*. **(A) **C57BL/6 mice hind footpads were infected with 3 x 10^6^ late stationary phase *L. guyanensis *parasites and disease progression was monitored by measuring footpad swelling over time. **(B)** Parasite load was quantified by *in vivo *imaging (Xenogen) measuring the luminescence signal (photon flux/10min/footpad). **(C)** C57BL/6 or **(D) **BALB/c mice hind footpads were infected with 3 x 10^6^ late stationary phase *L. major *parasites and disease progression was monitored by measuring footpad swelling over time. Results of a pool of 2 independent experiments **(A** and **B)** or one representative of 3 independent experiments **(C** and **D)** were expressed as mean ± SD (n ≥ 5). Statistical significance was assessed by *Repeated measure ANOVA ***(A **and** C) **or *Student’s t-test ***(B **and** D)**; ***p < 0.001, ns: non-significant.

To investigate whether polyP plays a role during *in vivo* infection we infected mouse footpads with *L. guyanensis* *VTC4* knockdown (StL#1) or *L. major* knockout (*vtc4^-^*) parasites and monitored footpad swelling on a weekly basis as a proxy for progression of the infection. The *L. guyanensis* M4147 WT line contains an integrated firefly luciferase gene (*LUC*), allowing parasite load to be quantified by bioluminescent imaging 48. We used the C57BL/6 mouse model, in which *L. guyanensis* induces self-healing footpad swelling 59. Infection with WT and the *GFP*-StL transfectant induced similar footpad swelling and parasite burden (Fig. 7A, B), peaking around 30 days post-infection and healing a few weeks later. *VTC4* knockdown with StL#1 showed little effect, with clone 22 showing a profile identical to WT and clone 21 showing a small, statistically not significant, effect. Thus, a 5- to 10-fold reduction in polyP levels (Fig. 2C) produced little effect on parasite infectivity (Fig. 7A and B).

For *L. major*, we used the self-healing C57BL/6 as well as the susceptible BALB/c mouse models. *vtc4^-^* *L. major* infected mice showed an important delay of several weeks before developing footpad lesions in the C57BL/6 as well as the BALB/c model (Fig. 7C and D). However, in all infections, parasites finally replicated and infectious parasites could be recovered from the footpad. Even though the complemented *vtc4^-^*/+VTC4 cell line recovered polyp synthesis, this did not restore its ability to rapidly induce lesions in mice (data not shown), thus preventing us from confirming a role of polyP in the course of the infection. The cells might have adapted to the loss of *VTC4* by second site mutations or genome rearrangements, leading to suppressor mutants that behave differently from the original WT.

### Absence of VTC4 and polyP lead to an increased sensitivity to heat shock

**Figure 8:**
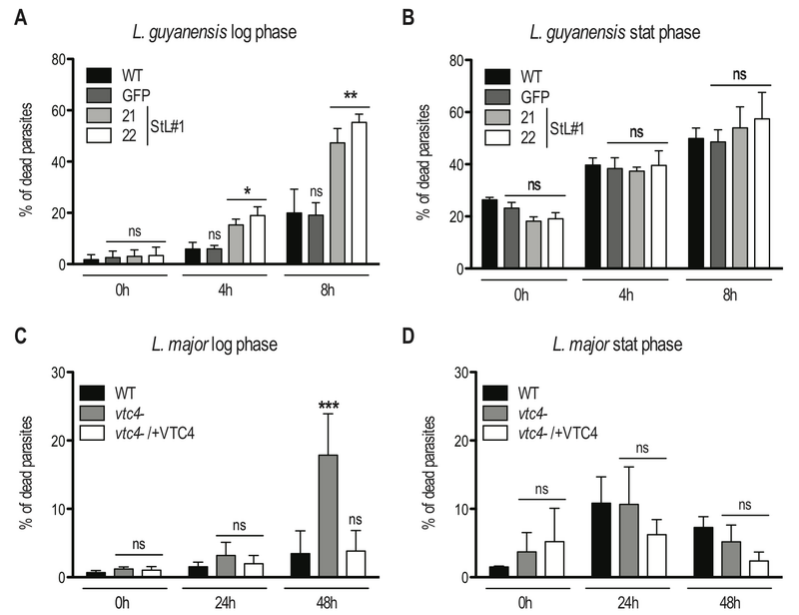
FIGURE 8: Lack of VTC4 and polyP results in increased cell death upon heat exposure. Logarithmic phase (day 3 post dilution) or stationary phase (day 6 post dilution) promastigotes of *L. guyanensis ***(A** and **B)** and *L. major* **(C** and **D)** were incubated at different temperatures (26°C and 37°C). The percentage of dead cells was assessed at the indicated time points by uptake of propidium iodide (PI). Results of a pool of 3 independent experiments were expressed as mean ± SD. Statistical significance was assessed by *Two-way ANOVA ***(A - D)**; * p < 0.05, ** p < 0.01, *** p < 0.001, ns: non-significant.

Next, we investigated whether polyP has an impact on parasite survival under different stress conditions encountered by *Leishmania* parasites during its life cycle, such as sudden increases of temperature. Parasites were grown in complete medium at 26°C and then split into two groups, of which one was exposed to 37°C, while a control was kept at 26°C. Parasite resistance was monitored by measuring cell death via propidium iodide staining at different time points. To better assess the effect of different polyP levels, both logarithmic growth phase (day 3 post dilution) and late stationary phase (day 6 post dilution) promastigotes were used. Presence of polyP increased the resistance to heat stress (37°C) of the polyP-rich logarithmic phase promastigotes from both *L. major* and *L. guyanensis* (Fig. 8A and C). PolyP-deficient *Leishmania* displayed an approximate 3-fold increase in cell death compared to controls, highlighting a protective role of polyP before parasites reached the stationary phase. By contrast, no significant effect on cell survival was observed in stationary phase promastigotes (Fig. 8B, D), which contain low amounts of polyP (Fig. 3B). Interestingly, *L. guyanensis* was much more sensitive to heat stress than *L. major, *with all clones already showing an increased frequency of cell death after 8 hours, while *L. major* was not affected during the first 24 hours.

**Figure 9:**
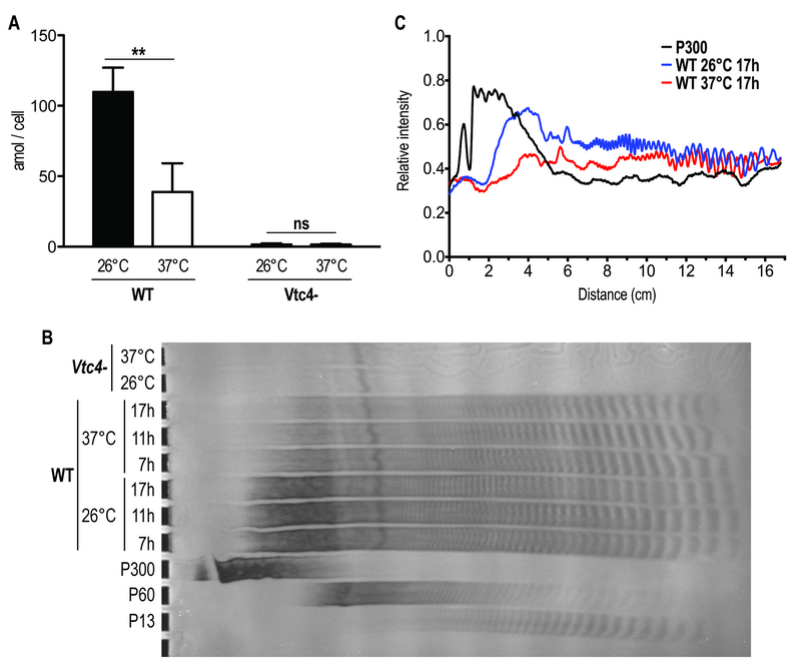
FIGURE 9: Higher temperature increases *Leishmania* consumption of polyP. **(A)** *L. major* logarithmic phase (day 3 post dilution) promastigotes were incubated for 6 hours at 26°C or 37°C. PolyP was quantified in attomole/cell (amol/cell) and represented as absolute values. Results of a pool of 3 independent experiments were expressed as mean ± SD of polyP levels. Statistical significance was assessed by *Two-way ANOVA*; ** p < 0.01, ns: non-significant. **(B)** PolyP was extracted from logarithmic phase (day 3 post dilution) *L. major *promastigotes exposed at different temperatures for the indicated time. PolyP chains were separated by gel electrophoresis and visualized by negative DAPI staining on a 35% polyacrylamide gel. PolyP standards represent an average of the respective sizes ranging from 13 up to 300 residues (P13, P60 and P300). **(C)** Quantification of polyP chains consumption by ImageJ of lanes of Fig. 9B representing polyP chains in *Leishmania* parasites cultured at 26°C (blue line) or exposed to a 37°C heat shock for 17h (red line). The black line represents the P300 standard and was included for comparison. The *x* axis represents the distance from the top of the gel and the *y* axis the relative intensity of the signal.

We quantified polyP in heat-treated logarithmic phase promastigotes and observed that, when maintained for several hours at 37°C, polyP became less abundant and the length of the polymer chains decreased in comparison to parasites cultured at 26°C (Fig. 9A, B), suggesting that longer-chain polyP is consumed under these conditions. A quantification of lanes of using ImageJ (Fig. 9C) showing a decrease in length of longer-chain polyP supports this hypothesis. This strengthens the correlation between polyP level and resistance to temperature stress. These results are consistent with our previous observation that *L. major* *vtc4^-^* parasites still differentiated into amastigotes but survived less than WT within macrophages 30. Thus, some parasites are killed and survivors are blocked in their replication till they are fully adapted to their new milieu. Once differentiated into amastigotes, our data showed that parasites do not require polyP to survive and to replicate in their host.

## DISCUSSION

Our study suggests that *Leishmania* VTC4 synthesizes long-chain polyP and that likely no other enzyme complements for this function. This differs from *T. brucei*, where VTC4 only provides a portion of the short-chain polyP since the conditional *VTC4* knockout of the blood stream form (BSF) resulted only in a 35% decrease in short-chain polyP but no significant changes in long-chain polyP 36. Furthermore, *vtc4^-^ L. major* promastigotes could be cultured *in vitro*, indicating that VTC4, and thereby also polyP, are not essential at this stage. In contrast*,* *T. brucei* conditional *VTC4* knockout BSF shows a progressive decrease in growth rate relative to the WT 36. These differences between trypanosomatids in terms of polyP regulation and function could be related to the fact that *T. brucei* resides extracellularly in both its insect and mammalian hosts, whereas *Leishmania* parasites proliferate inside the mammalian macrophage and possibly acquire phosphate from the host cell. Albeit present in all the *Leishmania* species analyzed, polyP abundance varies substantially between them (Fig. 1), underscoring the different synthesis abilities or consumption of this polymer.

Similar to previous observations in bacteria 60 and yeast 4,61, we found that polyP pools were highest at the end of the logarithmic phase and started to decrease once *Leishmania *promastigotes reached the stationary phase. Moreover, in the intracellular amastigote form, we detected only low amounts of polyP and VTC4, suggesting that, once the infection was established, amastigotes might not require high steady-state amounts of polyP to survive in their host. This could possibly explain why *VTC4* knockdown or knockout cell lines are still able to establish an infection *in vivo*. This observation differs from what was previously observed in *T. brucei, *where infection with conditional *VTC4 *knockout parasites induced lower parasitemia in the blood and 50% less death in BALB/c mice 36. In this latter case, the authors propose that infectivity is impaired through an increased susceptibility to osmotic shock, which results from a lack of polyP in *Trypanosoma* 37,42,62,63. As opposed to BSF *Trypanosoma*, *Leishmania* thrives intracellularly and osmoprotective roles of polyP might hence be less relevant in *Leishmania* than in Trypanosomes.

From the different stress conditions that we tested on *Leishmania *promastigotes (e.g. H_2_O_2_, phosphate buffered saline in the presence or absence of glucose and low pH) (data not shown), only heat shock at 37°C showed a polyP-related effect. PolyP-deficient *L. major* and polyP-low *L. guyanensis* were both more sensitive to increased temperature than the respective WT controls. However, the mechanism by which polyP promotes survival at high temperature remains unclear. Studies reported a rapid increase in cytosolic Ca^2+^ concentrations during physiological heat shock at 34 and 37°C, and suggest that this Ca^2+^ uptake is required for promastigote thermotolerance and differentiation into amastigotes 64,65. Since polyP complexes cations in acidocalcisomes 35,39,66, it might be required to store the absorbed Ca^2+^ that could then be released upon heat shock and increase thermotolerance. An alternative possibility is provided by the chaperone-like effects of polyphosphates, which counteract protein inactivation and aggregation upon heat stress 10. However, experiments on the solubility of proteins between WT and *vtc4-* parasites did not provide any evidence for such a possibility (H. Z and N.F, unpublished results).

The intracellular proliferation of *Leishmania* in the mammalian host comes with specific challenges, and polyP could play an important role in the first hours of infection, perhaps to facilitate the transition phase upon phagocytosis, during which the parasite must adapt to a radically different environment. In fact, inside phagosomes, *Leishmania *are exposed to an acidic pH and have restricted access to numerous nutrients, including metal ions, such as Mg^2+^, which can limit parasite growth inside the phagolysosome 67,68,69. Recent studies in *Saccharomyces cerevisiae* showed that this organism relies on polyP in order to grow under conditions of limited bioavailability of Mg^2+^ 30. The polyP stored in the acidocalcisome-like vacuoles may then be used as an immobilized chelator that filters ions from the environment. Interestingly, also *Leishmania* promastigotes lacking VTC4 and polyP grow poorly in low-Mg^2+^ media 70, suggesting that also in this organism polyP might be involved in extracting Mg^2+^ from the environment or in establishing sufficiently large reserves to support proliferation for a number of divisions in metal-poor environments. This could be relevant upon phagocytosis by a macrophage, as we previously showed that *vtc4^-^* *Leishmania* proliferate significantly less than WT within macrophages 30.

It should also not be neglected that *Leishmania* resides in two hosts. We found that polyP levels were highest at the end of the proliferating promastigote phase, which physiologically occurs in the sand fly, suggesting that polyP might be required for survival in the insect gut or for preparing the parasite for its next host. For instance, sand flies are mostly resident in tropical and subtropical regions where temperatures can reach high values and fluctuate daily (e.g. 20 - 40°C in India and North Africa), while nutrient availability and osmolality in the sand fly gut are changing with the ingested food, obliging parasites to adapt to the changing environment 71. It would thus be useful to investigate whether polyP also plays a role in adaptation to stresses in the insect vector.

## MATERIALS AND METHODS

### Mouse work

All mice studies were approved by the Swiss Federal Veterinary Office (SFVO), under the authorization number 2113. Animal handling and experimental procedures were undertaken with strict adherence to ethical guidelines set out by the SFVO and under inspection by the Department of Security and Environment of the State of Vaud, Switzerland.

### Parasite strains and clones

Parasites strains used in this study are described in Table S1.

### *VTC4* knockout and complementation in *L. major*

*VTC4* alleles were successively disrupted by hygromycin and puromycin resistance genes respectively 30. These constructs were based on the pX63 hygromycin vector 72. The linear targeting regions were gel-purified before transfection. Complemented lines were obtained by homologous recombination into either the *VTC4* or the small subunit ribosomal RNA locus 30.

### Stem-Loop (StL) RNAi constructs for *GFP* and *L. guyanensis *M4147 *VTC4*

*GFP*-StL (pIR1SAT-*GFP*65-StL(b) (B4733)) has been described previously 50. Two different sequences were used to make the stem-loop constructs (StL) with different selection markers (*BSD* and *PAC*, respectively) (Fig. S2). StL#1 and StL#2 arise from nucleotides 193 - 788 and 867 - 1963 of the *L. guyanensis* M4147 *VTC4* gene respectively (GenBank accession number: MF572933). StL constructs were generated using Gateway technology. First, StL#1 and StL#2 were amplified with primers P6425/P6426 and P6429/P6430, respectively (Table S2), and cloned into the pCR^®^ 8/GW/TOPO^®^ vector (Invitrogen cat# K2520-02). These constructs were then used separately to transfer to the *Leishmania* StL destination vectors pIR1*BSD*-GW and pIR1*PAC*-GW respectively, using Clonase II to perform the LR recombination reaction (Invitrogen cat#12535-029) as described previously 48.

### *Leishmania* culture

*L. major* promastigotes were grown at 26°C in M199 medium (Invitrogen AG) complemented with 10% heat-inactivated fetal bovine serum (FBS, Seromed GmbH), 50 U/ml penicillin/streptomycin (Amimed), 40 mM Hepes (Amimed), 0.6 mg/L biopterin (Sigma) and 5 mg/L hemin (Sigma). All other *Leishmania* strains were grown at 26°C in freshly prepared Schneider's insect medium (Sigma) supplemented with 10% heat-inactivated fetal bovine serum (PAA), 10 mM Hepes (Amimed), 50 U/ml penicillin/streptomycin (Amimed), 0.6 mg/L biopterin (Sigma) and 5 mg/L hemin (Sigma). *L. mexicana* axenic amastigote were obtained as previously described 73. Parasites were cultured in the above described Schneider’s insect medium supplemented with 20% FBS, at pH 5.4, 34°C in 5% CO_2_.

### *Leishmania major *transfection

Logarithmic promastigotes were transfected following the Amaxa short protocol: 8 - 12 µg linear DNA in a volume of 5 µl H_2_O were used to transfect 2 x 10^7^ parasites. Parasites were centrifuged (3200 rpm, 10 min) and washed in PBS (1x). The cell pellet was resuspended in 100 µl T cell Nucleofection solution of Amaxa human T cell Nucleofactor kit (Lonza) and the corresponding DNA was added. This mixture was then transferred to the amaxa cuvette and electroporated using the Nucleofector® II device, program U-033. Transfected and electroporated WT control parasites were incubated at 26°C in 4 ml complete M199 overnight. After 24 hours in drug-free media the parasites were plated on semisolid media (1% BactoAgar in complete M199) containing the appropriate drug to select clonal lines. Drug concentrations for selections were 100 µg/ml hygromycin, 50 µg/ml puromycin or 25 µg/ml neomycin. Parasite colonies were recovered from the plates after approximately 10 - 15 days and grown in liquid culture media supplemented with the appropriate antibiotics. The second round of homologous recombination was performed twice in two independent experiments, in order to decrease the possibility of second site mutations. To ensure clonal cultures, drug resistant colonies from semi-liquid plates were further subcloned.

### *Leishmania guyanensis *transfection

The parasites were grown to mid-logarithmic phase, pelleted at 1300 x *g*, washed once with cytomix electroporation buffer (120 mM KCl, 0.15 mM CaCl_2_, 10 mM K_2_HPO_4_, 25 mM HEPES-KOH pH 7.6, 2 mM EDTA and 5 mM MgCl_2_) and resuspended in cytomix at a final concentration of 2 × 10^8^ cells/ml. For transfection, 10 μg of *Swa*I digested DNA was mixed with 500 μl of cells and electroporated twice in a 0.4 cm gap cuvette at 25 microF, 1400 V (3.75 kV/cm), waiting 10 sec in-between zaps. Following electroporation, cells were incubated at 26°C for 24 hours in drug-free media and then plated on semisolid media containing the appropriate drug to select clonal lines. The digested *Swa*I DNA fragment was integrated into the ribosomal RNA SSU locus. For selections using blasticidin deaminase (*BSD* gene) and puromycin (*PAC* gene) markers, parasites were plated on 10 μg/ml blasticidin, 30 μg/ml puromycin, respectively, together with 50 µg/ml nourseothricin for all plates (as the parental line expresses luciferase with *SAT* marker). Colonies normally appeared after 14 days, at which point they were recovered, grown to stationary phase in 1 ml and passaged with the appropriate drugs. The efficacy of *VTC4* knockdown was measured by RT-PCR using the following primers: *VTC4*: forward: TGCGAGAGAAATACGACCCC, reverse: ACGTCCTGTGGCAGAAAGAG; *KMP11*: forward: GCCTGGATGAGGAGTTCAACA, reverse: GTGCTCCTTCATCTCGGG (Table S2).

### Parasite protein extraction

Logarithmic and stationary phase *L. major* promastigotes were lysed using the following method: parasites were diluted in PBS (1x) buffer containing 1 mM OPA (o-phenanthroline), 100 µM E64 (trans-epoxysuccinyl-L-leucylamido (4-guanidino) - butane), 5 µg/ml Pepstatin A, 10 µg/ml Leupeptin, 10 µg/ml Aprotinin, 1 mM PMSF and lysed by freeze and thaw with 5 successive passages of 2 minutes at 40°C and in liquid nitrogen. Supernatants (soluble proteins) were saved and the pellets (insoluble proteins) resuspended in 100 µl buffer as described above. Both were stored at -80°C. Protein concentration was quantified using a BCA (bicinchoninic acid) protein assay reagent (Pierce Biotechnology) with bovine serum albumine (BSA) as standard.

### Immunoblotting

8% polyacrylamide sodium dodecyl sulfate gels were used to separate 20 - 30 µg of pellet and supernatant proteins obtained from cell lysates. Proteins were transferred to a nitrocellulose membrane (Whatman) by electroblotting, probed with an affinity-purified rabbit polyclonal anti-cd-*Lmj*VTC4 antibody (1:1000) and a secondary anti-rabbit antibody (1:2500), which was coupled to HRP (Horse Radish Peroxidase - Promega Corp.), and a mouse anti-α-tubulin antibody (clone B5-1-2, Sigma-Aldrich) was used as loading control. Blots were developed by enhanced chemiluminescence (ECL) detection (Amersham Biosciences).

### PolyP column extraction and colorimetric quantification

Unless otherwise stated, column polyP extractions were performed on 6 x 10^7^ logarithmic phase (day 3 post dilution) promastigotes, or in the case of *L. major* on 1 - 2 x 10^8 ^promastigotes. Cells were washed in PBS (1x) and lysed with 50 µl 1 M H_2_SO_4_ and 50 µl 4 M NaOH. 100 µl 1 M Tris-Malate buffer (pH 7.4) were added to the lysate in order to stabilize the compounds. After addition of 600 µl binding buffer (Qiagen PCR purification kit), the lysate was transferred to the column (Qiagen PCR purification columns), centrifuged (1 min, 13000 rpm) and washed twice with 750 µl washing buffer of the kit. After an additional step of centrifugation (1 min, 13000 rpm), polyP was eluted with 110 µl elution buffer from the kit and samples were stored at -20°C.

For quantification, isolated polyP was degraded by a polyphosphatase (recombinant *S. cerevisiae* Ppx1, expressed and purified from *E. coli*) and the released P_i_ was stained with malachite green. For this, the polyP extract was mixed 1:1 with the master mix (10 mM Tris pH 7, 3 mM MgCl_2_, 0.4 µg/ml Ppx1) in a 96-well flat bottom microtiter plate and incubated for 90 min at 37°C. For staining, a mix of 86 µl molybdate solution (34.6 mg/ml ammonium molybdate and 1.12% concentrated H_2_SO_4_ in H_2_O) and 64 µl malachite green solution (0.3 mg/ml malachite green and 3.5 mg/ml polyvinyl alcohol in H_2_O) were added to each sample and incubated for 4 min. For each sample, a negative control without Ppx1 was incubated in parallel to determine the basal P_i _level before the reaction. The absorbance was read at 595 nm with a Synergy microplate reader (BioTek). This method was adapted from 61.

### PolyP precipitation and gel electrophoresis

PolyP was phenol (pH 4.3, Sigma) - chloroform extracted and precipitated in 100% ethanol at -20°C overnight (adapted from 74). Ethanol was removed and the pellet resuspended in H_2_O. Different enzymes were added to digest RNA (recombinant RNAse, Roche), DNA (recombinant DNAse I, Roche) and Ppx1 (recombinant *S. cerevisiae* PPX expressed and purified from *E. coli*) at 37°C. PolyP was resolved using a 24 x 16 x 0.1-cm gel with 30 - 35% polyacrylamide (Acrylamide/Bis 19:1, SERVA) in Tris-Borate-EDTA buffer. Before loading the samples, gels were pre-run for 60 min at 3 mA. Samples were run at 3 mA overnight at 4°C, until the loading dye (30% glycerol, 0.5% bromophenol blue, 1 mM EDTA in H_2_O) front migrated to ½ - ¾ of the gel. Gels were stained with DAPI (1.5 g/L Tris base, 2% glycerol, 2 mg/L DAPI) for 45 - 60 min, and destained with the same buffer without DAPI for 30 - 45 min. Gels were exposed to a UV transilluminator for 5 - 20 min to induce photobleaching, after which photographs were taken 75.

### High temperature stress

Logarithmic growth phase (day 3 post dilution) or stationary phase (day 6 post dilution) promastigotes were centrifuged, washed with minimal media and resuspended in the corresponding media: complete M199 for *L. major* and complete Schneider’s for *L. guyanensis*. Parasites were grown in duplicates (in 24-well plates) at 26 or 37°C, and the number of dead cells was assessed at the indicated time points using propidium iodide (PI) staining. 200 µl of propidium iodide diluted in PBS to 5 ml were added to 20 ml of parasite culture and, after 3 - 4 minutes of incubation at room temperature, samples were analyzed by flow cytometry using a BD Accuri^TM^ C6 Cytometer (BD Biosciences).

### PNA agglutination assay

Agglutination assays were performed with 1 - 2 x 10^8^ late stationary phase (day 6 post dilution) parasites in 1 ml and 50 μg/ml peanut agglutinin (Sigma) 76. After 30 min incubation at room temperature in DMEM (Gibco), cells were separated by centrifugation: 10 min at 200 x *g* yielded the cells agglutinated with PNA (PNA^+^), and 10 min at 1990 x *g* yielded the free PNA^-^ metacyclic parasites. Both parasite fractions were washed once with 10 ml DMEM supplemented with 20 mM galactose, in order to separate agglomerates. Parasites were then resuspended in DMEM and counted.

### Ficoll gradient centrifugation

Late stationary phase (day 6 post dilution) parasites were washed, resuspended in 2 ml DMEM and transferred into a glass tube. 2 ml of 10% and 20% ficoll (Sigma F5415) diluted in DMEM were added to the bottom of the tube to form a gradient. After centrifugation at 2500 rpm for 15 min (without brake), the upper 2 phases were collected, without touching the interphase below, and washed. Parasites were resuspended in DMEM and counted 57.

### Mouse infection

Mice were purchased from Harlan (C57BL/6) or Charles River (BALB/c). At the onset of experiments female mice were around 6 weeks of age and maintained under conventional conditions in an animal facility. Late stationary *Leishmania *parasites were washed in PBS and 3 x 10^6^ parasites were injected subcutaneously in a volume of 50 µl PBS into the hind footpad of mice. The progression of infection was evaluated by measuring the footpad size with a Vernier caliper and footpad swelling was defined by subtracting the thickness of the uninfected footpad from the infected one.

Parasite load during *in vivo* infection with *L. guyanensis* M4147 expressing luciferase was analyzed using the In Vivo Imaging System (IVIS Lumina II, Xenogen) at the Cellular Imaging Facility (CIF, University of Lausanne). Mice were injected intra-peritoneally with 150 mg/kg D-luciferin 10 min before imaging and anesthetized with isoflurane during imaging. The photons emitted from mouse footpads during a period of 10 min were quantified using the LivingImage version 3.2 software (Caliper Life Science) 59. Parasite burden was expressed as total photon flux / 10 min emitted from *L. guyanensis* infected footpad lesions normalized against the background luminescence of uninfected footpads or the tail.

### Amastigote isolation from footpads

Mouse footpads were excised in a sterile environment. The skin was removed and the footpad fragmented. After homogenizing the fragments in presence of MEG 1x (5.5 mM Glucose, 0.2 mM EDTA in PBS) using a 7 ml homogenizer, the lysate was centrifuged (400 rpm, 5 min, 4°C) to remove debris. The cell containing supernatant was centrifuged (3000 rpm, 10 min, 4°C) and the pellet treated with 10 ml RCRB (168 mM NH_4_Cl in H_2_O) for 10 min on ice. Amastigotes were washed twice with MEG 1x, passed through a 40 µm as well as an 8 µm filter and counted.

## SUPPLEMENTAL MATERIAL

Click here for supplemental data file.

All supplemental data for this article are also available online at http://microbialcell.com/researcharticles/importance-of-polyphosphate-in-the-leishmania-life-cycle/.

## References

[1] Alvar J, Velez ID, Bern C, Herrero M, Desjeux P, Cano J, Jannin J, den Boer M (2012). Leishmaniasis worldwide and global estimates of its incidence.. PloS one.

[2] Dostalova A, Volf P (2012). Leishmania development in sand flies: parasite-vector interactions overview.. Parasit Vectors.

[3] Kornberg A (1999). Inorganic polyphosphate: a molecule of many functions.. Prog Mol Subcell Biol.

[4] Kulaev I, Vagabov V, Kulakovskaya T (1999). New aspects of inorganic polyphosphate metabolism and function.. J Biosci Bioeng.

[5] Brown MR, Kornberg A (2008). The long and short of it - polyphosphate, PPK and bacterial survival.. Trends Biochem Sci.

[6] Seufferheld MJ, Alvarez HM, Farias ME (2008). Role of polyphosphates in microbial adaptation to extreme environments.. Appl Environ Microbiol.

[7] Rao NN, Gomez-Garcia MR, Kornberg A (2009). Inorganic polyphosphate: essential for growth and survival.. Annu Rev Biochem.

[8] Kim KS, Rao NN, Fraley CD, Kornberg A (2002). Inorganic polyphosphate is essential for long-term survival and virulence factors in Shigella and Salmonella spp.. Proc Natl Acad Sci U S A.

[9] Kornberg A (1995). Inorganic polyphosphate: toward making a forgotten polymer unforgettable.. J Bacteriol.

[10] Gray MJ, Wholey WY, Wagner NO, Cremers CM, Mueller-Schickert A, Hock NT, Krieger AG, Smith EM, Bender RA, Bardwell JC, Jakob U (2014). Polyphosphate is a primordial chaperone.. Mol Cell.

[11] Wang L, Fraley CD, Faridi J, Kornberg A, Roth RA (2003). Inorganic polyphosphate stimulates mammalian TOR, a kinase involved in the proliferation of mammary cancer cells.. Proc Natl Acad Sci U S A.

[12] Hernandez-Ruiz L, Gonzalez-Garcia I, Castro C, Brieva JA, Ruiz FA (2006). Inorganic polyphosphate and specific induction of apoptosis in human plasma cells.. Haematologica.

[13] Schroder HC, Kurz L, Muller WE, Lorenz B (2000). Polyphosphate in bone.. Biochemistry Biokhimiia.

[14] Leyhausen G, Lorenz B, Zhu H, Geurtsen W, Bohnensack R, Muller WE, Schroder HC (1998). Inorganic polyphosphate in human osteoblast-like cells.. J Bone Miner Res.

[15] Shiba T, Nishimura D, Kawazoe Y, Onodera Y, Tsutsumi K, Nakamura R, Ohshiro M (2003). Modulation of mitogenic activity of fibroblast growth factors by inorganic polyphosphate.. J Biol Chem.

[16] Pavlov E, Aschar-Sobbi R, Campanella M, Turner RJ, Gomez-Garcia MR, Abramov AY (2010). Inorganic polyphosphate and energy metabolism in mammalian cells.. The J Biol Chem.

[17] Morrissey JH, Choi SH, Smith SA (2012). Polyphosphate: an ancient molecule that links platelets, coagulation, and inflammation.. Blood.

[18] Smith SA, Choi SH, Davis-Harrison R, Huyck J, Boettcher J, Rienstra CM, Morrissey JH (2010). Polyphosphate exerts differential effects on blood clotting, depending on polymer size.. Blood.

[19] Ishige K, Zhang H, Kornberg A (2002). Polyphosphate kinase (PPK2), a potent, polyphosphate-driven generator of GTP.. Proc Natl Acad Sci U S A.

[20] Achbergerova L, Nahalka J (2011). Polyphosphate--an ancient energy source and active metabolic regulator.. Microb Cell Fact.

[21] Ogawa N, DeRisi J, Brown PO (2000). New components of a system for phosphate accumulation and polyphosphate metabolism in Saccharomyces cerevisiae revealed by genomic expression analysis.. Mol Biol Cell.

[22] Freimoser FM, Hurlimann HC, Jakob CA, Werner TP, Amrhein N (2006). Systematic screening of polyphosphate (poly P) levels in yeast mutant cells reveals strong interdependence with primary metabolism.. Genome Biol.

[23] Hothorn M, Neumann H, Lenherr ED, Wehner M, Rybin V, Hassa PO, Uttenweiler A, Reinhardt M, Schmidt A, Seiler J, Ladurner AG, Herrmann C, Scheffzek K, Mayer A (2009). Catalytic core of a membrane-associated eukaryotic polyphosphate polymerase.. Science.

[24] Muller O, Bayer MJ, Peters C, Andersen JS, Mann M, Mayer A (2002). The Vtc proteins in vacuole fusion: coupling NSF activity to V(0) trans-complex formation.. Embo J.

[25] Muller O, Neumann H, Bayer MJ, Mayer A (2003). Role of the Vtc proteins in V-ATPase stability and membrane trafficking.. J Cell Sci.

[26] Gerasimaite R, Sharma S, Desfougeres Y, Schmidt A, Mayer A (2014). Coupled synthesis and translocation restrains polyphosphate to acidocalcisome-like vacuoles and prevents its toxicity.. J Cell Sci.

[27] Gerasimaite R, Pavlovic I, Capolicchio S, Hofer A, Schmidt A, Jessen HJ, Mayer A (2017). Inositol Pyrophosphate Specificity of the SPX-Dependent Polyphosphate Polymerase VTC.. ACS Chem Biol.

[28] Desfougeres Y, Gerasimaite RU, Jessen HJ, Mayer A (2016). Vtc5, a Novel Subunit of the Vacuolar Transporter Chaperone Complex, Regulates Polyphosphate Synthesis and Phosphate Homeostasis in Yeast.. J Biol Chem.

[29] Wild R, Gerasimaite R, Jung JY, Truffault V, Pavlovic I, Schmidt A, Saiardi A, Jessen HJ, Poirier Y, Hothorn M, Mayer A (2016). Control of eukaryotic phosphate homeostasis by inositol polyphosphate sensor domains.. Science.

[30] Klompmaker SH, Kohl K, Fasel N, Mayer A (2017). Magnesium uptake by connecting fluid-phase endocytosis to an intracellular inorganic cation filter.. Nat Commun.

[31] Uttenweiler A, Schwarz H, Neumann H, Mayer A (2007). The vacuolar transporter chaperone (VTC) complex is required for microautophagy.. Mol Biol Cell.

[32] Fang J, Rohloff P, Miranda K, Docampo R (2007). Ablation of a small transmembrane protein of Trypanosoma brucei (TbVTC1) involved in the synthesis of polyphosphate alters acidocalcisome biogenesis and function, and leads to a cytokinesis defect.. Biochem J.

[33] Lemercier G, Espiau B, Ruiz FA, Vieira M, Luo S, Baltz T, Docampo R, Bakalara N (2004). A pyrophosphatase regulating polyphosphate metabolism in acidocalcisomes is essential for Trypanosoma brucei virulence in mice.. J Biol Chem.

[34] Moreno B, Urbina JA, Oldfield E, Bailey BN, Rodrigues CO, Docampo R (2000). 31P NMR spectroscopy of Trypanosoma brucei, Trypanosoma cruzi, and Leishmania major. Evidence for high levels of condensed inorganic phosphates.. J Biol Chem.

[35] Docampo R, Jimenez V, Lander N, Li ZH, Niyogi S (2013). New insights into roles of acidocalcisomes and contractile vacuole complex in osmoregulation in protists.. Int Rev Cell Mol Biol.

[36] Lander N, Ulrich PN, Docampo R (2013). Trypanosoma brucei Vacuolar Transporter Chaperone 4 (TbVtc4) Is an Acidocalcisome Polyphosphate Kinase Required for in Vivo Infection.. J Biol Chem.

[37] Ruiz FA, Rodrigues CO, Docampo R (2001). Rapid changes in polyphosphate content within acidocalcisomes in response to cell growth, differentiation, and environmental stress in Trypanosoma cruzi.. J Biol Chem.

[38] Docampo R, Moreno SN (2011). Acidocalcisomes.. Cell Calcium.

[39] Docampo R, de Souza W, Miranda K, Rohloff P, Moreno SN (2005). Acidocalcisomes - conserved from bacteria to man.. Nat Rev Microbiol.

[40] Docampo R, Jimenez V, King-Keller S, Li ZH, Moreno SN (2011). The role of acidocalcisomes in the stress response of Trypanosoma cruzi.. Adv Parasitol.

[41] Besteiro S, Tonn D, Tetley L, Coombs GH, Mottram JC (2008). The AP3 adaptor is involved in the transport of membrane proteins to acidocalcisomes of Leishmania.. J Cell Sci.

[42] de Jesus TC, Tonelli RR, Nardelli SC, da Silva Augusto L, Motta MC, Girard-Dias W, Miranda K, Ulrich P, Jimenez V, Barquilla A, Navarro M, Docampo R, Schenkman S (2010). Target of rapamycin (TOR)-like 1 kinase is involved in the control of polyphosphate levels and acidocalcisome maintenance in Trypanosoma brucei.. J Biol Chem.

[43] Galizzi M, Bustamante JM, Fang J, Miranda K, Soares Medeiros LC, Tarleton RL, Docampo R (2013). Evidence for the role of vacuolar soluble pyrophosphatase and inorganic polyphosphate in Trypanosoma cruzi persistence.. Mol Microbiol.

[44] Madeira da Silva L, Beverley SM (2010). Expansion of the target of rapamycin (TOR) kinase family and function in Leishmania shows that TOR3 is required for acidocalcisome biogenesis and animal infectivity.. Proc Natl Acad Sci U S A.

[45] Rodrigues CO, Ruiz FA, Vieira M, Hill JE, Docampo R (2002). An acidocalcisomal exopolyphosphatase from Leishmania major with high affinity for short chain polyphosphate.. J Biol Chem.

[46] Altschul SF, Gish W (1996). Local alignment statistics.. Methods Enzymol.

[47] Waterhouse AM, Procter JB, Martin DM, Clamp M, Barton GJ (2009). Jalview Version 2 - a multiple sequence alignment editor and analysis workbench.. Bioinformatics.

[48] Lye LF, Owens K, Shi H, Murta SM, Vieira AC, Turco SJ, Tschudi C, Ullu E, Beverley SM (2010). Retention and loss of RNA interference pathways in trypanosomatid protozoans.. PLoS pathogens.

[49] Peacock CS, Seeger K, Harris D, Murphy L, Ruiz JC, Quail MA, Peters N, Adlem E, Tivey A, Aslett M, Kerhornou A, Ivens A, Fraser A, Rajandream MA, Carver T, Norbertczak H, Chillingworth T, Hance Z, Jagels K, Moule S, Ormond D, Rutter S, Squares R, Whitehead S, Rabbinowitsch E, Arrowsmith C, White B, Thurston S, Bringaud F, Baldauf SL (2007). Comparative genomic analysis of three Leishmania species that cause diverse human disease.. Nat Genet.

[50] Robinson KA, Beverley SM (2003). Improvements in transfection efficiency and tests of RNA interference (RNAi) approaches in the protozoan parasite Leishmania.. Mol Biochem Parasitol.

[51] Atayde VD, Shi H, Franklin JB, Carriero N, Notton T, Lye LF, Owens K, Beverley SM, Tschudi C, Ullu E (2013). The structure and repertoire of small interfering RNAs in Leishmania (Viannia) braziliensis reveal diversification in the trypanosomatid RNAi pathway.. Mol Microbiol.

[52] Rooney PJ, Ayong L, Tobin CM, Moreno SN, Knoll LJ (2011). TgVTC2 is involved in polyphosphate accumulation in Toxoplasma gondii.. Mol Biochem Parasitol.

[53] Ulrich PN, Lander N, Kurup SP, Reiss L, Brewer J, Soares Medeiros LC, Miranda K, Docampo R (2013). The Acidocalcisome Vacuolar Transporter Chaperone 4 Catalyzes the Synthesis of Polyphosphate in Insect-stages of Trypanosoma brucei and T. cruzi.. J Eukaryot Microbiol.

[54] McConville MJ, Turco SJ, Ferguson MA, Sacks DL (1992). Developmental modification of lipophosphoglycan during the differentiation of Leishmania major promastigotes to an infectious stage.. EMBO J.

[55] Turco SJ, Descoteaux A (1992). The lipophosphoglycan of Leishmania parasites.. Annu Rev.

[56] Sacks DL (2001). Leishmania-sand fly interactions controlling species-specific vector competence.. Cell Microbiol.

[57] Spath GF, Beverley SM (2001). A lipophosphoglycan-independent method for isolation of infective Leishmania metacyclic promastigotes by density gradient centrifugation.. Exp Parasitol.

[58] Blum JJ (1989). Changes in orthophosphate, pyrophosphate and long-chain polyphosphate levels in Leishmania major promastigotes incubated with and without glucose.. J Protozool.

[59] Ives A, Ronet C, Prevel F, Ruzzante G, Fuertes-Marraco S, Schutz F, Zangger H, Revaz-Breton M, Lye LF, Hickerson SM, Beverley SM, Acha-Orbea H, Launois P, Fasel N, Masina S (2011). Leishmania RNA virus controls the severity of mucocutaneous leishmaniasis.. Science.

[60] Rao NN, Liu S, Kornberg A (1998). Inorganic polyphosphate in Escherichia coli: the phosphate regulon and the stringent response.. J Bacteriol.

[61] Werner TP, Amrhein N, Freimoser FM (2005). Novel method for the quantification of inorganic polyphosphate (iPoP) in Saccharomyces cerevisiae shows dependence of iPoP content on the growth phase.. Arch Microbiol.

[62] Fang J, Ruiz FA, Docampo M, Luo S, Rodrigues JC, Motta LS, Rohloff P, Docampo R (2007). Overexpression of a Zn2+-sensitive soluble exopolyphosphatase from Trypanosoma cruzi depletes polyphosphate and affects osmoregulation.. J Biol Chem.

[63] Huang G, Fang J, Sant'Anna C, Li ZH, Wellems DL, Rohloff P, Docampo R (2011). Adaptor protein-3 (AP-3) complex mediates the biogenesis of acidocalcisomes and is essential for growth and virulence of Trypanosoma brucei.. J Biol Chem.

[64] Sarkar D, Bhaduri A (1995). Temperature-induced rapid increase in cytoplasmic free Ca2+ in pathogenic Leishmania donovani promastigotes.. FEBS letters.

[65] Naderer T, Dandash O, McConville MJ (2011). Calcineurin is required for Leishmania major stress response pathways and for virulence in the mammalian host.. Mol Microbiol.

[66] Huang G, Bartlett PJ, Thomas AP, Moreno SN, Docampo R (2013). Acidocalcisomes of Trypanosoma brucei have an inositol 1,4,5-trisphosphate receptor that is required for growth and infectivity.. Proc Natl Acad Sci U S A.

[67] Garcia-del Portillo F, Foster JW, Maguire ME, Finlay BB (1992). Characterization of the micro-environment of Salmonella typhimurium-containing vacuoles within MDCK epithelial cells.. Mol Microbiol.

[68] Mann FM, VanderVen BC, Peters RJ (2011). Magnesium depletion triggers production of an immune modulating diterpenoid in Mycobacterium tuberculosis.. Mol Microbiol.

[69] Eriksson S, Lucchini S, Thompson A, Rhen M, Hinton JC (2003). Unravelling the biology of macrophage infection by gene expression profiling of intracellular Salmonella enterica.. Mol Microbiol.

[70] Lanza H, Afonso-Cardoso SR, Silva AG, Napolitano DR, Espindola FS, Pena JD, Souza MA (2004). Comparative effect of ion calcium and magnesium in the activation and infection of the murine macrophage by Leishmania major.. Biol Res.

[71] Lefurgey A, Gannon M, Blum J, Ingram P (2005). Leishmania donovani amastigotes mobilize organic and inorganic osmolytes during regulatory volume decrease.. J Eukaryot Microbiol.

[72] Cruz A, Coburn CM, Beverley SM (1991). Double targeted gene replacement for creating null mutants.. Proc Natl Acad Sci U S A.

[73]  Bates  PA (1994). Complete developmental cycle of Leishmania mexicana in axenic culture.. Parasitology.

[74] Lonetti A, Szijgyarto Z, Bosch D, Loss O, Azevedo C, Saiardi A (2011). Identification of an evolutionarily conserved family of inorganic polyphosphate endopolyphosphatases.. J Biol Chem.

[75] Smith SA, Morrissey JH (2007). Sensitive fluorescence detection of polyphosphate in polyacrylamide gels using 4',6-diamidino-2-phenylindol.. Electrophoresis.

[76] Dasilva R, Sacks DL (1987). Metacyclogenesis Is a Major Determinant of Leishmania Promastigote Virulence and Attenuation.. Infect Immun.

