# Supplementary material for: Importance of polyphosphate in the Leishmania life cycle
Source: Microb Cell. 2018 Jun 22;5(8):371–84. doi: 10.15698/mic2018.08.642 (PMC6116282; doi:10.15698/mic2018.08.642)
Supplement: Supplementary file 1 [file mic-05-371-s01.pdf]

Supplementary Information

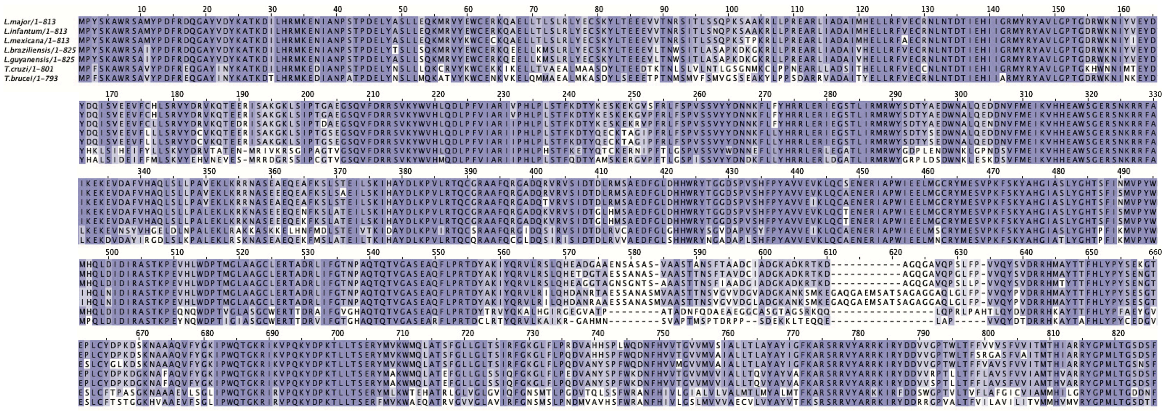

Fig. S1. Amino acid sequence comparison of *Leishmania* and *Trypanosoma* VTC4.

A multiple alignment between the different VTC4 amino acid sequences was performed, using ClustalW alignment, within Jalview**.** The degree of sequence similarity is represented in a dark blue to white color code, indicating high to low sequence conservation of amino acid residues, respectively. *Lmj*: *L. major*; *Lin*: *L. infantum*; *Lmex: L. mexicana*; *Lbr: L. braziliensis; L.gy: L. guyanensis; Tc: T. cruzi; Tb: T. brucei.* The accession numbers of the various sequences used in this study are the following: *L. major*: Q4QI04, GN=LmjF09.0220; *L. infantum*: A4HTX5, GN=LinJ09.0590; *L. mex*: E9AMM1, GN=LmxM09.0220; *L. braziliensis*: A4H5J7, GN=LbrM09_V2.0220; *L. guyanensis*: MF572933; *T. cruzi*: Q4DRN6, GN=Tc00.1047053509023.100; *T. brucei*: Q382V9, GN=Tb11.01.4040.

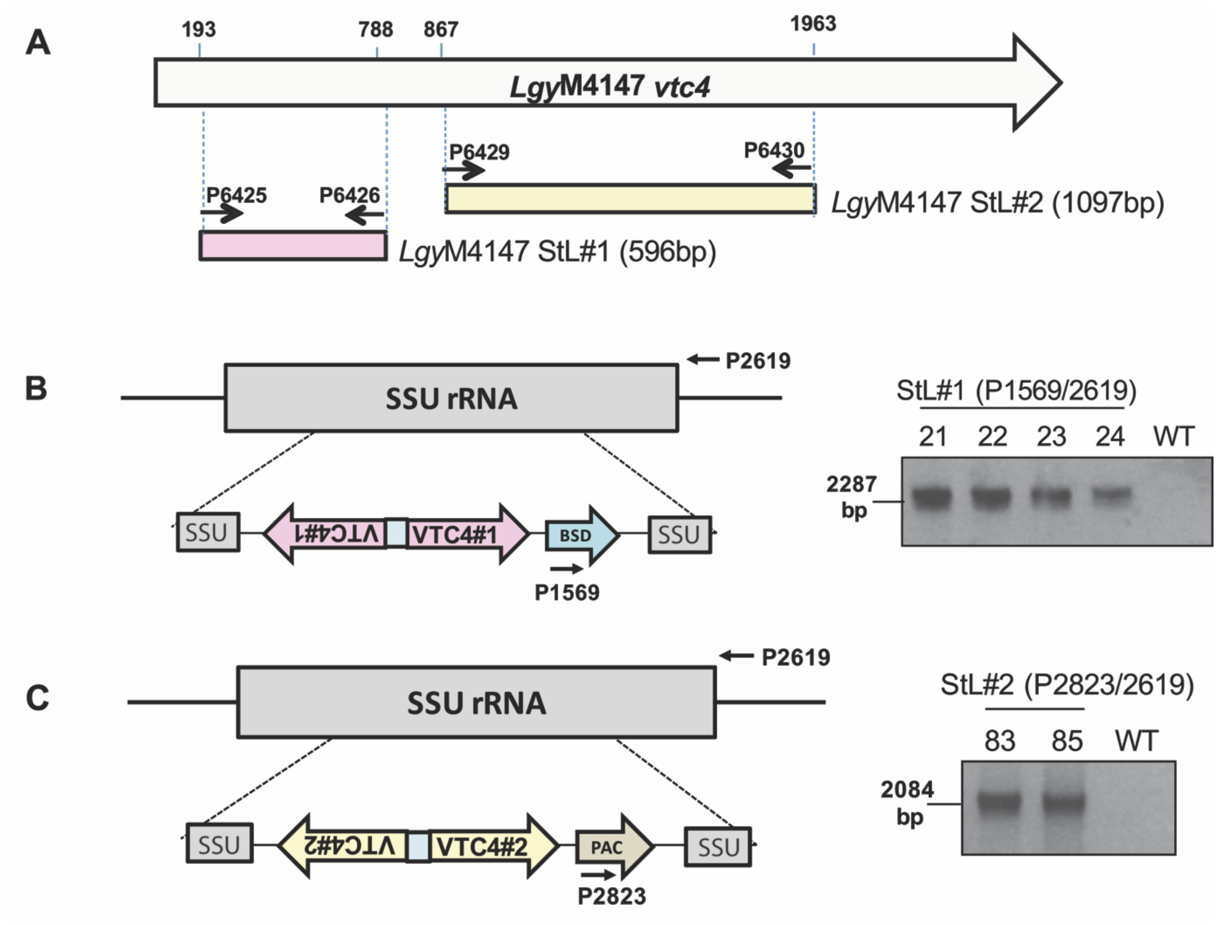

Fig. S2. RNAi interference on *VTC4* in *L. guyanensis* parasites.

(A) Schematic map of StL#1 targeting the 5' end of the gene (193-788 bp) and StL#2 targeting the center (867- 1963 bp) of *L. guyanensis VTC4*. **(B, C)** Genomic integration of constructs for stem loop formation was verified by PCR on genomic DNA. Amplification at the expected size was detected for primers P1569/P2619 in **(B)** StL#1 and for primers P2823/P2619 in **(C)** StL#2 transfectants. Primer sequences are presented in Table S2.

Table S1. *Leishmania* species and clones used.

Species	Strain
L. major	MRHO/IR/75/ER (IR75) and MRHO/SU/59/P (LV39 clone 5)
L. guyanensis	MHOM/BR/75/M4147 and WHI/BR/78/M5313
L. mexicana mexicana	MYNC/BZ/62/M379
L. donovani donovani	MOHOM/IN/1983/AG83
L. aethiopica	MHM/ET/2008/LDS372
L. braziliensis	MHOM/BR/00/LTB325
L. panamensis	MHOM/ CO/86/1166

Table S2. Primers used for *L. guyanensis VTC4* knockdown generation and for qRT-PCR.

Primer	Sequence (5'→3')	Note
P6425	CGCTAGTCTAGA**aagcaggaggagttgctgaaga**	under line: XbaI site; bold: *VTC4* sequences
P6426	CGCTAGTCTAGA**acggagctgacgggcgagaaga**	under line: XbaI site; bold: *VTC4* sequences
P6429	CGCTAGTCTAGA**gtggtactcggacacgtactcc**	under line: XbaI site; bold: *VTC4* sequences
P6430	CGCTAGTCTAGA**agtaggggtagaggtggaaggt**	under line: XbaI site; bold: *VTC4* sequences
P1569	TATGATACTAGTATGACCGAGTACAAGCCCAC	forward BSD
P2619	CGACTTTTGCTTCCTCTATTG	Rev SSU
P2823	GGAGGAAGATCTCCACCATGGCCAAGCCTTTGTCTCAAG	forward PAC
fw qRT-KMP11	GCCTGGATGAGGAGTTCAACA	qRT-PCR forward primer for *KMP11* gene
rv qRT-KMP11	GTGCTCCTTCATCTCGGG	qRT-PCR reverse primer for *KMP11* gene
fw qRT-Lg-VTC4	TGCGAGAGAAATACGACCCC	qRT-PCR forward primer for *VTC4* gene
fw qRT-Lg-VTC4	ACGTCCTGTGGCAGAAAGAG	qRT-PCR reverse primer for *VTC4* gene
